# Organization of distributed cortical connections underlying the processing of auditory information in dogs assessed by diffusion MRI

**DOI:** 10.1162/IMAG.a.16

**Published:** 2025-05-28

**Authors:** Mélina Cordeau, Sophie A. Barton, Erin E. Hecht

**Affiliations:** Department of Human Evolutionary Biology, Harvard University, Cambridge, MA, United States

**Keywords:** auditory processing, communication, domestication, dog, diffusion MRI, neuroanatomy, structural connectivity, evolutionary biology

## Abstract

Dogs and humans have co-evolved for millennia. This provides an opportunity to examine neural adaptations supporting cross-species communication. Previous canine fMRI studies have identified functional activations in response to human voice perception. However, the specific neural pathways involved in dogs’ ability to process and respond to human language remain unknown. This study takes a data-driven approach to examine the brain connectivity supporting bidirectional communication in a large sample of dogs. We examine white matter pathways linking temporal regions, involved in the perception of communicative signals, and frontal regions, responsible for generating communicative responses. Using cortical regions with known axonal connectivity from tract tracing studies as a foundation, we applied probabilistic tractography to measure connectivity patterns in a diverse cohort of dogs (n = 110, 16 breeds). Our findings reveal that, beyond short-range intra-regional connections, consistent large-scale tracts connect the prefrontal, somatosensory, premotor, motor, and temporal lobes across subjects. Hierarchical clustering analysis revealed distinct structural organization, with sylvian regions strongly connected to motor regions and ectosylvian regions linked to higher-order frontal and prefrontal regions. This organization may suggest that the ectosylvian gyrus plays a key role in integrating auditory input with complex cognitive functions, potentially underlying cross-species communication and language processing in dogs. This study elucidates cortico-cortical communication pathways in dogs and contributes to our understanding of the neural basis of lexical processing in the canine brain.

## 1 Introduction

Spoken language as we know it is a uniquely human ability. One of the major challenges in neuroscience is understanding how language emerged and evolved, and how it is supported by its underlying neural mechanisms. Domestic dogs, having evolved alongside humans, may have undergone positive selection for the capacity to understand some aspects of human language. This historical selection sets them apart from other animal species, including non-human primates.

Humans have selected dogs for various specialized abilities—guarding, hunting, protection, or simple companionship—which has led to significant differences in behavior and trainability in different breed lineages (Serpell & Hsu, 2005;Tonoike, 2015). Furthermore, recent research suggests that the ability to understand human communication is the result of specific evolutionary selection (Byosiere et al., 2023).

Neural processing of human vocalizations appears to rely on specific regions and circuits. Neuroimaging studies have shown that regions such as the caudal ectosylvian and sylvian gyri are selectively activated by familiar verbal stimuli (Andics et al., 2014;Cuaya et al., 2022;Gábor et al., 2020), supporting a form of semantic representation. In rare cases, like Chaser the Border Collie, elements of combinatorial understanding have also been reported (Pilley, 2013).

Previous studies have investigated neural variation in dogs, including the effects of skull shape on brain morphology (Barton et al., 2024;Carreira & Ferreira, 2015;Pilegaard et al., 2017), anatomical correlates of specific behaviors (Jacobs et al., 2007;Våge et al., 2010), variation in brain organization across breed lineages in relation to temperament and historical working behaviors (Hecht et al., 2019;Hecht, Zapata, et al., 2021), and anatomical asymmetry in the canine brain (Barton et al., 2023). Furthermore, fMRI studies have identified brain regions sensitive to words that dogs understand (Gábor et al., 2020). Additional work has shown that dogs can also detect novel words, offering a behaviorally grounded perspective on neural processing (Prichard et al., 2018). Electrophysiological research has pinpointed frontal cortex regions that, when electrically stimulated, prompt vocalization in dogs (Kirikae et al., 1962;Krause, 1884;Milojevic & Hast, 1964). Additionally, Kosmal’s tract-tracing work (Kosmal, 1981a,1981b,^2000^;Kosmal & Dabrowska, 1980;Kosmal et al., 1984,^2004^;Malinowska & Kosmal, 2003;Markow-Rajkowska & Kosmal, 1987;Rajkowska & Kosmal, 1988,^1989^) has mapped the anatomical connections of the canine subcortical, temporal, and frontal lobes. Despite these advancements, a quantitative assessment of the organization of connectivity among these regions remains lacking.

It is likely that selection pressure for the ability to engage in interspecies communication has led to complex, structured patterns of white matter connectivity between cortical areas associated with vocal perception and higher-order brain regions responsible for generating appropriate responses to human vocal communication. Domestic dogs offer a unique opportunity to examine how domestication may have influenced neuroanatomy related to communication.

Therefore, the present study adopted a fully data-driven approach, encompassing the entire auditory network to (i) validate that dMRI tractography can recapitulate known direct axonal connections that were previously identified in tract-tracing studies and (ii) assess the higher-order organization of distributed communication networks using clustering analyses.

## 2 Materials and Methods

All procedures were reviewed and approved by the Institutional Animal Care and Use Committee (IACUC) and the Institutional Review Board (IRB) at Harvard University.

### 2.1 Data

We studied n = 110 dogs from different breeds: Border Collie (11), German Shepherd (15), German Shorthair Pointer (1), Indian Village Dog (1), Korean Village Dog (1), Kishu Ken (2), Labrador Retriever (55), Golden Retriever (2), mixed breed (1), New Guinea Singing Dog (1), Saluki (1), Samoyed (2), Shiba Inu (1), Siberian Husky (2), Sprint Racing Alaskan Sled Dog (13), and West Siberian Laika (1). The sample included both males (n = 53) and females (n = 57), with a mean age of 3.2 years (SD = 2.19, range: 0.58 to 11 years). While factors such as age and sex were not explicitly modeled in the current analyses, the relatively large and heterogeneous sample was designed to be representative of the broader canine population, thus capturing a wide range of variability in brain organization. However, given that some breeds were represented by only one or two individuals, breed-specific interpretations should be made with caution.

### 2.2 Anesthesia

Procedures occurred under the supervision of a veterinarian. Dogs were sedated with butorphanol and dexmedetomidine (0.3 mg/kg + 3-5 mcg/kg IM). Anesthesia was induced with propofol (to effect, 2-6 mg/kg IV) and maintained with isoflurane 100% O_2_. Dogs were continuously monitored under anesthesia, including ECG, SpO_2_, NIBP (oscillometric), and capnography, using an InVivo Expression MRI-safe unit.

### 2.3 MRI sequences

Scans were acquired using a 3.0 Tesla Siemens MAGNETOM Prisma whole-body MRI system in Harvard University’s Center for Brain Science, outfitted with a Siemens 15-channel TX/RX knee coil (Siemens Healthineers, Erlangen, Germany). Images included T1-weighted images (0.67 mm^3^, 2 averages), T2-weighted structural images (0.67 mm^3^, 1 average), and diffusion-weighted images (1.30 mm^3^, 60 diffusion directions, 2 averages acquired with reversed phase encoding, 12 B0s). Scan duration was approximately 1 h per dog. After the scan was complete, dogs were recovered from anesthesia, returned to their owners, and sent home with a framed image of their brain and a bandana.

### 2.4 Image processing

The FSL software package was used for preprocessing and analysis (Jenkinson et al., 2012;Smith et al., 2004). Study-specific T1, T2, and FA templates were created using the ANTS buildtemplateparallel.sh script, which creates an unbiased average of inputted image sets (Avants et al., 2014). These templates were created in alignment with the Cornell canine brain template (Johnson et al., 2020). Each subject’s diffusion data were processed as follows. EPI distortion was corrected using TOPUP (Andersson et al., 2003), eddy currents were corrected using EDDY (Andersson & Sotiropoulos, 2016), diffusion tensors were fit using DTIFIT (T. e. j. Behrens et al., 2003,^2007^), and BEDPOSTX was used to generate a probabilistic distribution of fiber population orientations (T. e. j. Behrens et al., 2003,^2007^)

### 2.5 Experimental design and statistical analyses

#### 2.5.1 ROIs

**Fig. 1.**
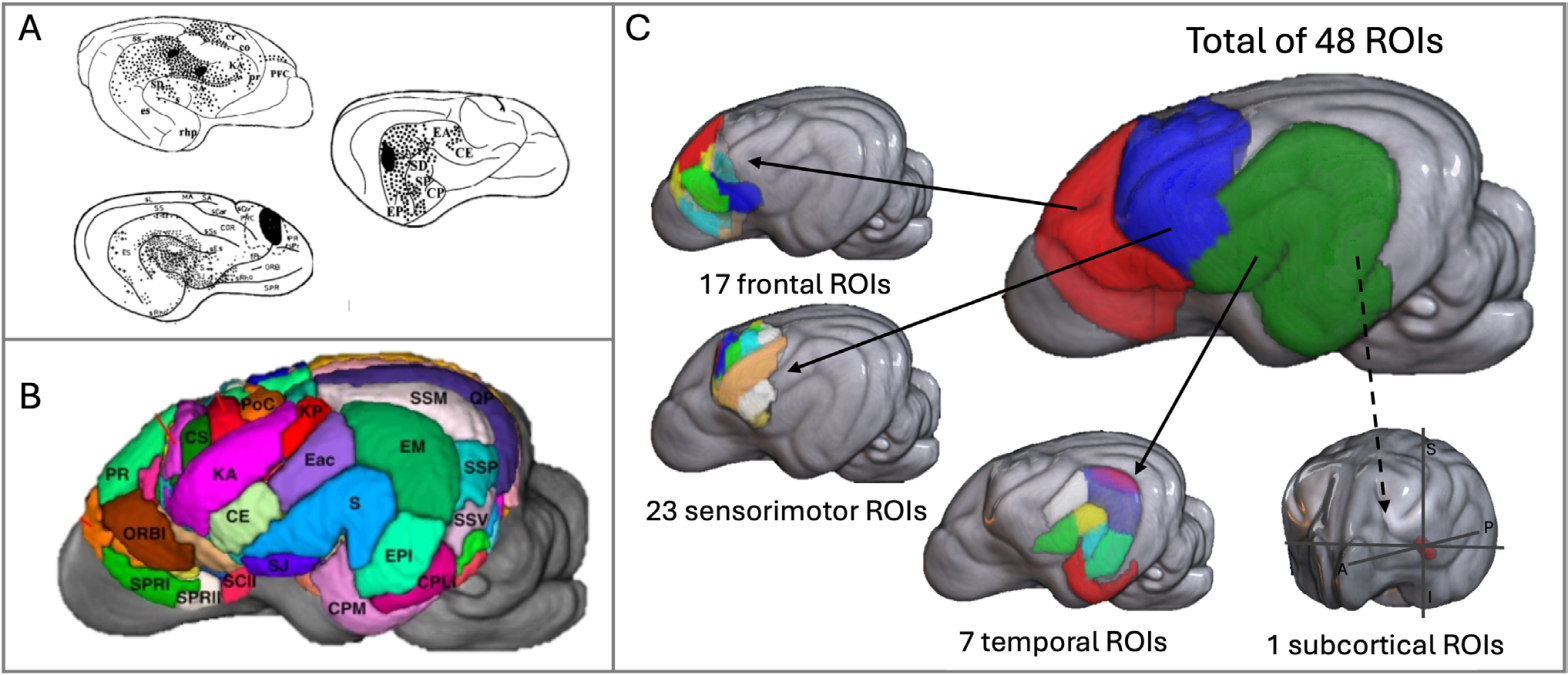
Approach used to generate the regions of interest (ROIs). (A) Example of visualizations from the tract tracer studies conducted byKosmal (2000)used to analyze anatomical projections of the auditory network. (B) 3D representation of the Johnson atlas used in our dMRI study. (C) Regions of the Johnson atlas selected to cover all areas with anatomical projections to the auditory cortex, according to Kosmal.

The ROIs were selected from Cornell cortical and subcortical atlas (Johnson et al., 2020;Fig. 1B), informed by connectivity patterns observed in prior tract-tracing studies (Kosmal, 2000;Kosmal et al., 1984;Markow-Rajkowska & Kosmal, 1987). These tracing studies demonstrated structural connectivity between regions involved in auditory processing (Fig. 1A). Therefore, we extracted temporal, sensorimotor, premotor, and prefrontal cortical regions from the atlas that were identified by prior tract-tracing studies to exhibit direct afferent or efferent axonal connectivity with the auditory cortex (Fig. 1C).

SeeAnnexe 1for the table of selected ROIs. A total of 48 regions were chosen: 17 frontal, 23 sensory–motor, 7 temporal, and 1 subcortical. Subsequently, these regions were projected into the subject space using nonlinear diffeomorphic transforms computed by ANTS, and binarized. Probtrackx2 was used for tractography (T. e. j. Behrens et al., 2003,^2007^).

*Connectivity matrices*were performed using the Pandas library (1.2.4, (McKinney, 2022)) for each subject individually and for each hemisphere. Visualization of the connectivity matrix was carried out using Seaborn’s heatmap function (0.11.1 (Waskom, 2021)) and Matplotlib (v3.3.4 (Caswell et al., 2021)). Each matrix illustrates pairwise connections between all regions. All matrices were individually normalized for the tractography waytotal count so that each had a number of connections between 0 and 100, and each connection was normalized relative to the size of the ROIs (in mm^3^, see the Table inAnnexe 1). An average of the 110 matrices was computed and is depicted inFigure 2A. The strongest connections were highlighted using stars for the top 1%, 5%, and 10% of connectivity values.*Histograms*were generated to visualize and compare the distribution of connections between each lobe (temporal, prefrontal, sensorimotor, premotor, and motor). Each axis of the histogram represents the average proportion of connections normalized by individual and by ROI size (data from the previously shown connectivity matrix) on a logarithmic scale, allowing for a direct comparison of the directions taken by connections across different lobes. This approach, in addition to the connection values provided by the matrix, offers an overview of the distribution of these connections between the lobes.

**Fig. 2.**
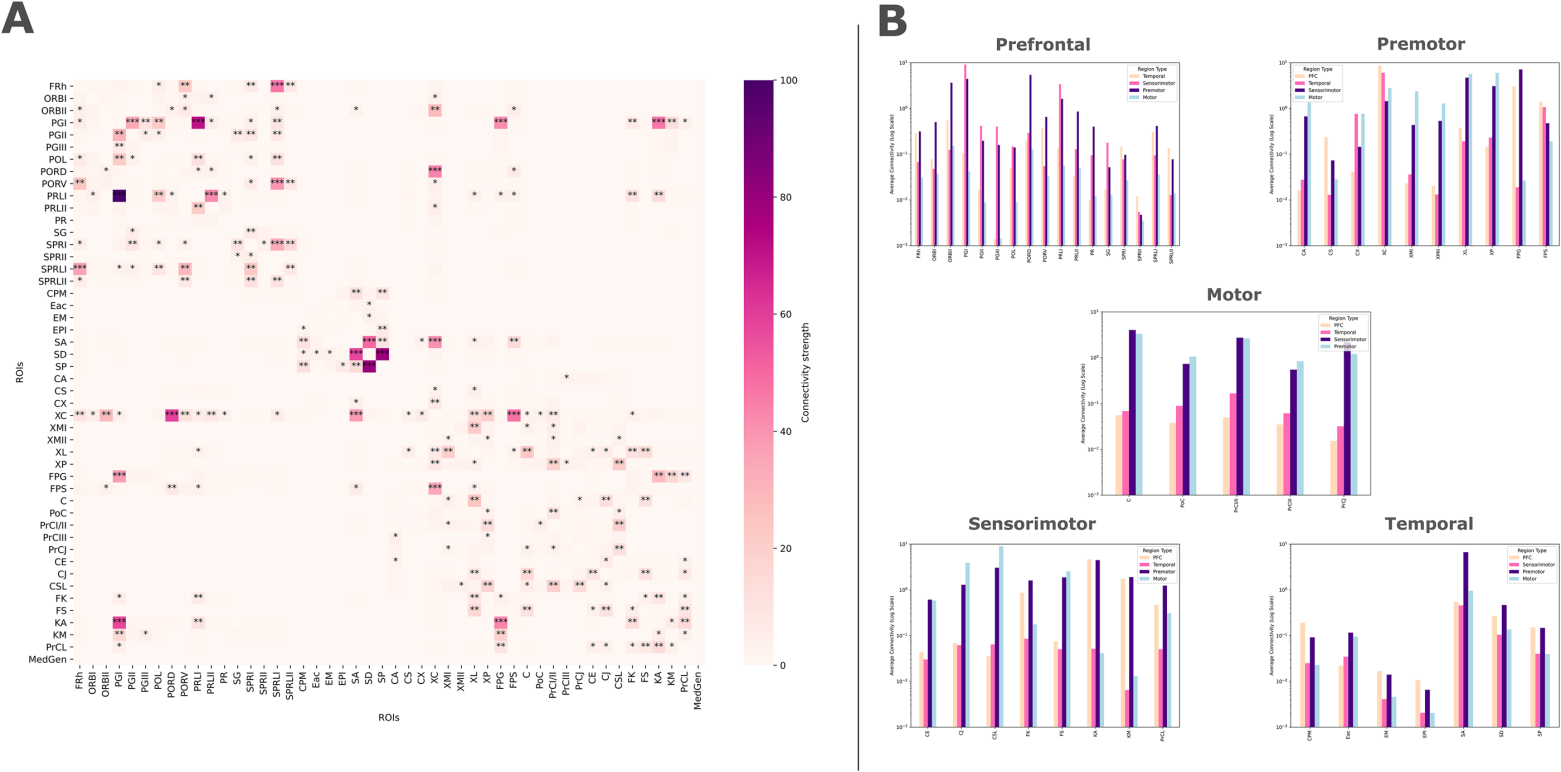
Connectivity matrix and description of projections from each region to others. (A) The matrix was normalized for the number of individual waytotal and for the ROIs size. The color bar denotes the strength of connection between two regions. Stars indicate the top 1%, 5%, and 10% of the strongest connectivity values, as operationalized by waytotal counts, between regions. (B) Histograms represent the average connections from each region to other lobes. The scale is logarithmic, and the different colors represent the various lobes.

Our method was validated by comparing it with canine tract-tracing studies as a “ground truth”. After reviewing these studies (Kosmal, 1981a,1981b,^2000^;Kosmal & Dabrowska, 1980;Kosmal et al., 1984,^2004^;Malinowska & Kosmal, 2003;Markow-Rajkowska & Kosmal, 1987;Rajkowska & Kosmal, 1988,^1989^), we constructed a matrix based on the literature, which we then compared with our matrix obtained through tractography analysis. We analyzed the common connections as well as the false positives and false negatives present in our results.

#### 2.5.2 Hierarchical cluster analysis

Using a hierarchical clustering approach (Ward method, implemented in the seaborn.clustermap function from the Seaborn library v0.11.1 (Waskom, 2021) and data preprocessing conducted using the Pandas library v1.2.4 (McKinney, 2022)), the dendrogram was generated from the pairwise connectivity matrix between brain regions. Cutoff thresholds were defined to allow a clear visualization of the grouping of regions based on their similarities. Colors associated with the thresholds were then used to highlight the clusters represented on the surfaces. For the tractography inFigure 4C, it was performed using the FSL tool probtrackx2 (T. E. J. Behrens et al., 2007) with the network parameter to display only the tracts connecting the ROIs of interest. The tractography was thresholded at the individual level to remove the bottom 1% of the waytotal number, then binarized and projected onto the template before being concatenated across all subjects. A threshold was then applied again at the group level to visualize above-threshold connectivity in 90% of the subjects.

## 3 Results

### 3.1 Structural connectivity of the auditory processing network in dogs

The matrix inFigure 2Arepresents the average of 220 individual structural connectivity matrices (110 left and 110 right), with regions selected from the Johnson atlas in pairwise fashion. The stars represent the strongest connections. This highlights that most of the strongest connections occur locally within the frontal, temporal, and sensorimotor lobes (along the diagonal). We also observe a smaller number of strong, longer-distant connections between these lobes. Notably, the strong connections from the temporal lobe to the frontal and sensorimotor lobes originate from the Sylvian Gyrus (SA). Additionally, other strong connections interlink the prefrontal, sensorimotor, premotor, and motor areas. A visual representation of this connectivity matrix is provided inFigure 5. The histograms inFigure 2Brepresent the average connections from each region of one lobe to another. This chart allows us to visualize the distribution of connections across all the lobes studied. We observe that the prefrontal cortex has many connections to the sensorimotor and premotor areas, though some regions also have numerous connections to the temporal lobe. Premotor regions primarily project to the sensorimotor and motor areas, with some also connecting to the temporal lobe. For motor regions, it is mainly the sensorimotor and premotor areas that project into this lobe. The sensorimotor lobe is more diverse: some regions have stronger affinities with the premotor and motor areas, while others are more connected to the prefrontal cortex. Finally, within the temporal lobe, one region primarily projects to the premotor area, while the remaining temporal regions are versatile, balancing their projections across the PFC, premotor, motor, and sensorimotor areas.

### 3.2 Validation of the results based on tract-tracing studies

Continuing our analysis, we aimed to verify the validity of the matrix we generated against the ground truth from previous studies using tract tracing (Kosmal, 1981a,1981b,^2000^;Kosmal & Dabrowska, 1980;Kosmal et al., 1984,^2004^;Malinowska & Kosmal, 2003;Markow-Rajkowska & Kosmal, 1987;Rajkowska & Kosmal, 1988,^1989^). Therefore, we created a matrix based on the literature, following the same configuration as our structural connectivity matrix, to allow for a comparison between the two. The matrix based on the literature is depicted inFigure 3A. The matrix we presented inFigure 2is shown again inFigure 3B. The matrix depicted inFigure 3Cis the test matrix we have generated, which we “cleaned” using the tract-tracing results, meaning we removed connections from the dMRI matrix if prior tract-tracing literature established a lack of connectivity between those two regions (i.e., false positives). From this matrix, we determined our false positives and negatives related to the tractography technique (shown inFig. 3Dand3E, respectively).

**Fig. 3.**
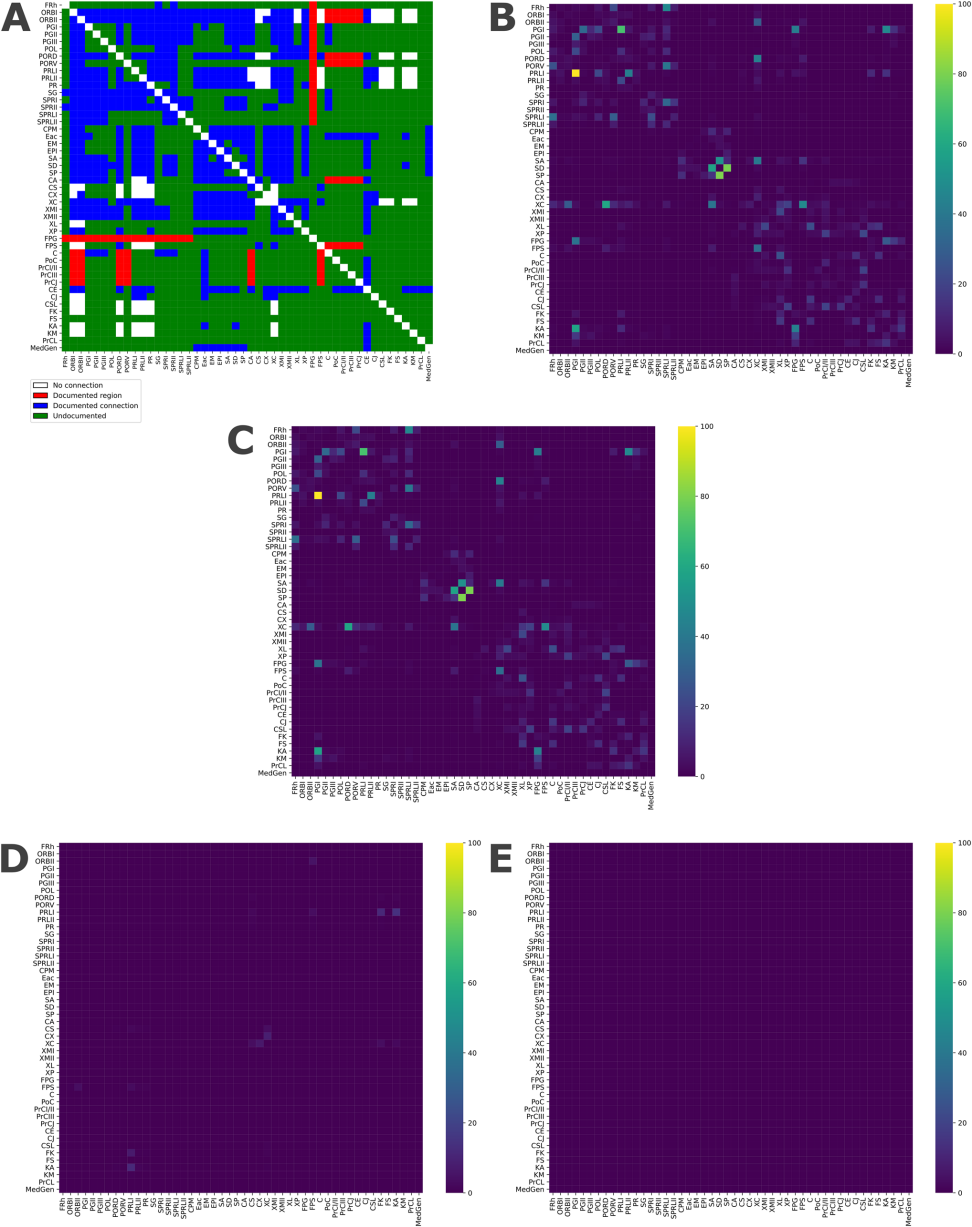
Structural connectivity matrix validation.**(**A) Connectivity matrix based on prior tract-tracing studies. We used different colors for this matrix: white when the connection was shown to be nonexistent, red when the region was shown to be involved, blue when the connection was previously demonstrated, and green when the connection had not yet been documented; (B) matrix generated by our tractography method; (C) matrix generated by our tractography method and cleaned with the ground truth matrix in A; (D) false positive results from our matrix; (E) false negative results from our matrix.

### 3.3 Hierarchical organization to observe structural connectivity

**Fig. 4.**
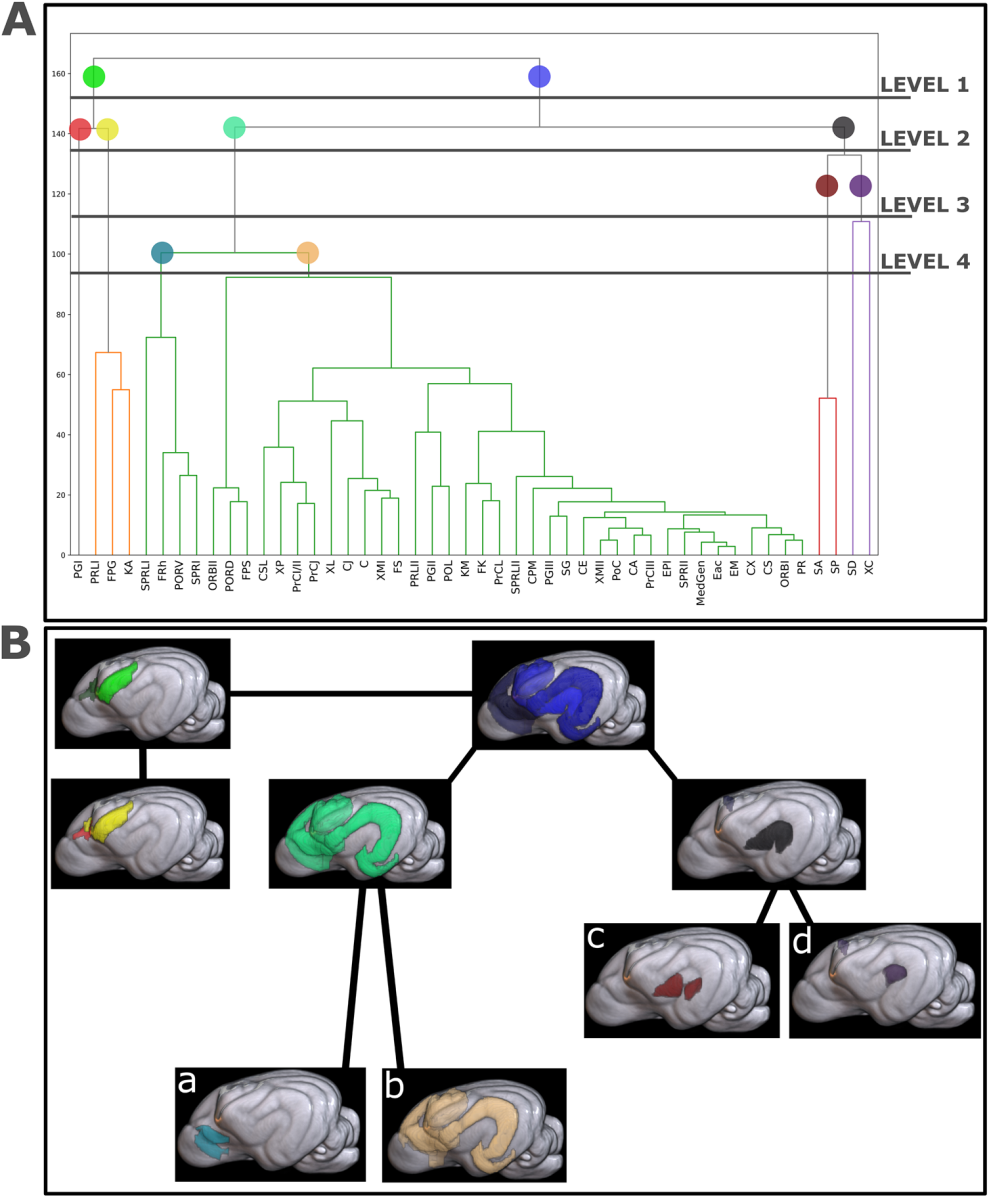
Hierarchical cluster analysis. (A) The dendrogram presented here was created from the average connectivity matrix, which was generated from the tractogram of 110 dogs as shown inFigure 2B. The 48 cortical regions are represented on the x-axis, and 4 levels are illustrated on the y-axis to represent the formation and composition of clusters. (B) Clusters are represented on the canine cortical surface for visualization. The colors of dendrogram markers in A correspond to the colors of clusters in B.

The dendrogram was generated from the average connectivity matrix (Fig. 2A) using a hierarchical clustering method to group structural connections of similar strength. This approach allowed the identification of both networks and sub-networks within the data. For our analysis, we divided the dendrogram into four levels to distinguish the two main networks and their sub-networks (Fig. 4A).Figure 4Bprovides a cortical surface projection of the dendrogram.

At the first level, a distinction was observed between somatosensory regions (green) and fronto-temporal regions (blue). At level 2, the fronto-temporal regions further subdivided into two distinct subgroups: one comprised the ectosylvian gyrus clustered with the prefrontal, premotor, and motor cortices, while the other included the sylvian gyrus, which clustered with the premotor regions. At level 3, the sylvian gyrus further split into two sections, with the SA-SP regions remaining closely interconnected (Fig. 4B.c), and the SD region showing stronger connectivity with the premotor areas (Fig. 4B.d). At level 4, the prefrontal, premotor, motor, and ectosylvian regions continued to cluster together (pale orange figureFig. 4B.b), while a portion of the prefrontal cortex formed a separate sub-cluster (light blue figureFig. 4B.a).

### 3.4 Organization of distributed cortical connections underlying the processing of auditory information

**Fig. 5.**
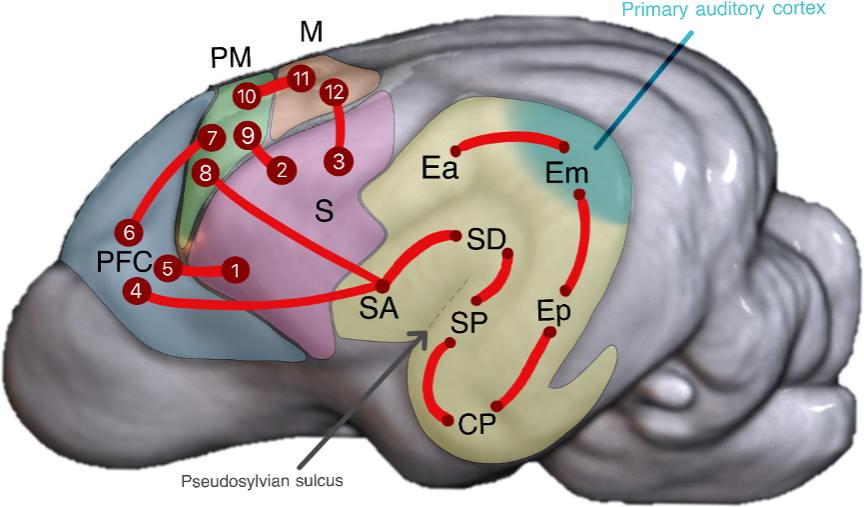
New model of structural connectivity in the auditory processing of dogs. Figure adapted fromKosmal (2000). Em: middle ectosylvian area; Ep: posterior ectosylvian area; Ea: anterior ectosylvian area; SD: dorsal sylvian area; SP: posterior sylvian area; SA: anterior sylvian area; CP: posterior composite area; S: sensory area; PFC: prefrontal cortex; PM: premotor area; M: motor area. The numbers correspond to cortical regions:**1:**FK (Area fissurae coronalis), KA (Area coronalis anterior), KM (Area coronalis medialis), PrCL (Area precentral lateralis);**2:**CE (Area composita ectosylvia), CSL (Area composita sigmoidea lateralis), FK (Area fissurae coronalis), FS (Area fissurae splenialis), KA (Area coronalis anterior), KM (Area coronalis medialis), PrCL (Area precentral lateralis);**3:**CSL (Area composita sigmoidea lateralis), FS (Area fissurae splenialis);**4:**ORBII (Area orbitalis II);**5:**PGI (Area pregenualis I), PGIII (Area pregenualis III), PRLI (Area Prorealis lateralis I);**6:**FRh (Area fissurae orbitalis), ORBI (Area orbitalis I), ORBII (Area orbitalis II), PGI (Area pregenualis I), PORD (Area paraorbitalis dorsalis), PORV (Area paraorbitalis ventralis), PRLI (Area Prorealis lateralis I), PRLII (Area Prorealis lateralis II), PR (area prorealis), SPRLI (Area Subprorealis lateralis I);**7:**XC (Area precruciata centralis), XL (Area precruciata lateralis), FPG (Area fissurae pregenualis), FPS (Area fissurae presylviae);**8:**CX (Area composita precruciata), XC (Area precruciata centralis), FPS (Area fissurae presylviae), XL (Area precruciata lateralis);**9:**XC (Area precruciata centralis), XMII (Area precruciata medialis II), XL (Area precruciata lateralis), XP (Area precruciata posterior), FPG (Area fissurae pregenualis), CJ (Area composita interna);**10:**CA (Area composita anterior), XC (Area precruciata centralis), XMI (Area precruciata medialis I), XMII (Area precruciata medialis II), XL (Area precruciata lateralis), XP (Area precruciata posterior), CJ (Area composita interna);**11:**C (Area centralis), PoC (Area postcentralis I), PrCI/II (Area precentralis I/II), PrCIII (Area precentralis III), PrCJ (Area precentralis interna);**12:**C (Area centralis), PoC (Area postcentralis I), PrCI/II (Area precentralis I/II), PrCJ (Area precentralis interna).

Figure 5illustrates inter-regional structural connections identified through dMRI from the average connectivity matrix of 110 subjects (Fig. 5). Connectivity between pairs of regions is depicted by red lines. Region abbreviations are detailed in the legend. This model demonstrates temporal connectivity as previously shown byKosmal (2000). Additionally, the diagram displays connections from somatosensory regions to motor, premotor, and prefrontal regions, providing detailed information about the specific regions within these larger areas. We highlighted a unique connection between the temporal lobe and frontal lobe, linking SA to ORBII. We observed that SA, which is a temporal region, contributes to connections extending toward the PM cortex. Premotor and motor regions are interconnected, while the PFC connects with premotor, temporal, and sensory regions. It should be noted that this figure summarizes the inter-regional connections identified as the strongest in the connectivity matrix, corresponding to at least the top 10% of connection strengths. Some weaker connections are not represented here but are, of course, still present.

## 4 Discussion

In this study, we used dMRI probabilistic tractography in 110 dogs to examine the organization of distributed cortico-cortical connectivity between auditory cortex and somatosensory, premotor, motor, and prefrontal regions. This analysis was motivated by the unique selection pressures and lifetime learning experiences that dogs, as co-evolved companions of humans, have undergone for interspecies communication.

To ensure the robustness of our diffusion MRI analyses, we adopted an approach aimed at reducing false positives. False positives are common in dMRI due to noise or partial volume effects (Alexander et al., 2001). To address this, we constrained our analysis to verified axonal connections established by prior tract-tracing studies, ensuring the consistency and accuracy of our approach. This process eliminated the few weak false positives which occurred (seeFig. 3D). dMRI largely replicated prior tract-tracing results (Fig. 3), confirming its utility in mapping canine cortical networks.

Our analysis then focused on comparing diffusion MRI tractography results with previous findings. Auditory information reaches the temporal cortex through the medial geniculate nucleus of the thalamus. Consistent with previous tract-tracing studies (Kosmal, 2000;Malinowska & Kosmal, 2003), we observed strong interconnections between the medial geniculate nucleus and the middle ectosylvian gyrus (EM) and ectosylvian area (EA). Weaker connections with the SA, SD, and SP regions were also noted, aligning with Kosmal et al.’s findings (Kosmal et al., 2004). These results align with the role of EM as the primary auditory cortex, functionally analogous to the human superior temporal cortex (Cuaya et al., 2022).

From EM, auditory information progresses to higher-order regions in the ectosylvian and sylvian gyri (Kosmal, 2000). fMRI studies in awake dogs show that these regions process complex auditory information (Andics et al., 2014;Boros et al., 2020;Cuaya et al., 2022;Reinholz-Trojan et al., 2012) and form distributed networks integrating auditory inputs with other cortical functions. Our dMRI analysis quantified the relative strength of these connections and their hierarchical organization through clustering.

The study of connectivity matrices and clustering analysis provided insights into the organization of connections. The connectivity matrix (Fig. 2A) and histogram (Fig. 2B) provide overviews of inter-regional brain connections, highlighting the strongest ones (Fig. 5). While direct fronto-temporal connections appeared weak in the matrix, the histogram revealed extensive, dispersed connections between these lobes. Consistent withKosmal’s (2000)observations, the temporal lobe connects broadly to premotor, motor, prefrontal, and sensorimotor regions, positioning it as a central hub for auditory processing and integration with higher-level functions.

Hierarchical clustering identified two major clusters: one grouping the sylvian region (SD) with the premotor cortex, and another linking the ectosylvian regions with prefrontal, premotor, motor, and sensorimotor cortices (Fig. 4). Notably, SD and XC exhibited extensive, diverse connections to all lobes, reflecting their central role. SA and SP, strongly connected to SD, formed a distinct subgroup. These patterns suggest that sylvian regions are closely interconnected within the auditory–motor system, consistent with structural covariation findings (Hecht et al., 2019) that align with genetic and developmental specialization (Alexander-Bloch et al., 2013;Evans, 2013).

Examining the functional implications of the identified connections allows us to assess their role in multisensory integration and social cognition. The connections of somatosensory cortex with temporal, prefrontal, premotor, and motor cortices highlight its role in multisensory integration, essential for coordinated behaviors (Guran et al., 2024). Our histogram (Fig. 2B) supportsKosmal’s (2000)identification of ventral and anterior networks based on anatomical location. However, given the evolutionary divergence between primates and carnivores (Kaas, 2019;Kumar et al., 2017), functional analogies to human auditory pathways should be approached cautiously.

Research suggests that in dogs, natural and artificial selection for understanding human communication has shaped brain regions involved in speech perception. Functional neuroimaging identifies canine temporal regions, including the caudal ectosylvian and sylvian gyri, as key for linguistic representation (Andics et al., 2014;Cuaya et al., 2022;Gábor et al., 2020;Prichard et al., 2018). These regions, connected to prefrontal, premotor, motor, and somatosensory areas, likely support high-level integrative processing essential for social cognition. In particular, the sylvian and prorean gyri are implicated in social behavior, with structural modifications linked to domestication (Hecht, Zapata, et al., 2021;Kukekova et al., 2011).

The prorean gyrus’ enlargement in fossil records correlates with enhanced social communication in pack hunting (Radinsky, 1969). Structural covariation between the prorean and sylvian gyri predicts traits such as fear and aggression toward strangers (Hecht et al., 2019;Hecht, Kukekova, et al., 2021). Furthermore, dog breeds that have undergone historical selection primarily for companionship as opposed to working functions (i.e., “lap dogs”) show gray matter morphological changes in the sylvian and prorean gyri (Hecht et al., 2019). In the Russian farm-fox experiment, selection for friendly social behavior toward humans is also associated with gray matter morphology enlargement in the prorean gyrus, and a network which includes parts of the sylvian gyrus covaries with social behavior toward humans (Hecht et al., 2021). These findings suggest a multisensory integration role for the sylvian gyrus, which clusters with the vocal premotor cortex (XC). While dogs do not engage in complex vocal imitation as humans do, this region may nonetheless support sensorimotor integration for socially relevant vocal cues, potentially contributing to the mapping of auditory input onto contextually appropriate behavioral responses.

Although awake dog fMRI studies face limitations in examining prefrontal regions due to EPI distortions, lesion studies suggest that the prefrontal cortex modulates inhibitory responses (Allen, 1949;Cook et al., 2016;Dabrowska & Szafrańska-Kosmal, A., 1972). This region’s connections with temporal auditory areas likely support multimodal integration for guiding social interactions. In addition to the roles of the prorean gyrus in canine social behavior noted above, tame foxes also show gene expression differences in the prorean gyrus (Kukekova et al., 2011). Taken together, this evidence suggests that the auditory networks identified by the present analysis may play important roles in hereditary and experience-dependent variations in interspecies communication and perception in canine domestication.

Premotor and somatosensory regions also contribute to dogs’ responses to human communication. While dogs do not produce speech, premotor regions involved in the control of vocal tract muscles may still play a role in processing and generating socially relevant vocalizations such as barking or whining. These areas may also support broader sensorimotor functions related to interpreting and responding to communicative cues. Premotor areas are involved in processing linguistic stimuli, such as distinguishing familiar from unfamiliar language (Cuaya et al., 2022), and play a role in controlling vocal motor functions, including the movement of laryngeal and pharyngeal muscles as well as vocal fold adduction (Kirikae et al., 1962;Krause, 1884). Somatosensory regions, particularly the primary and secondary somatosensory areas (SI and SII), show lateralized activation depending on the side of tactile stimulation. Additionally, the canine anterior ectosylvian gyrus, homologous to human receptive speech areas, is selectively active during tactile, verbal, and combined social reinforcement tasks (Guran et al., 2024). This suggests that aspects of human–dog communication may have evolved from general mammalian brain structures specialized for social reinforcement (Miller et al., 2024). While auditory–motor and auditory–premotor connections are indeed present in other mammals, including rodents (Nakata et al., 2020), the question remains whether the organization and engagement of these circuits in response to human communicative signals have been shaped by domestication and coevolution. We do not assume that such pathways are entirely unique to dogs; rather, we hypothesize that their functional tuning and integration within socio-communicative contexts may have been enhanced through selection for interspecies communication. Comparative work with non-domesticated canids such as wolves and coyotes will be essential to assess the specificity and potential elaboration of these pathways in dogs.

Our use of the terms “dorsal” and “ventral” pathways in the canine brain refers primarily to anatomical connectivity patterns—respectively, the “anterior” and “ventral” pathways—identified in tract tracer studies (Kosmal, 2000), rather than to direct functional homologies with human language networks. With this anatomical framework in mind, we explored evolutionary and functional parallels to better understand similarities and differences with human auditory pathways. The dorsal auditory pathway in dogs, connecting the rostral ectosylvian gyrus to premotor cortex, is proposed to support sound localization (Kosmal, 2000). In humans, the dorsal pathway underlies sensorimotor aspects of communication, potentially including syntactic processing (Friederici, 2016;Saur et al., 2008). While dogs may not process syntax like humans, some have been argued to demonstrate aspects of syntactic understanding (Pilley, 2013). Regions in both canine dorsal and ventral pathways, including the ectosylvian gyrus, appear involved in semantic understanding, activated by familiar linguistic stimuli (Cuaya et al., 2022;Gábor et al., 2020). The last common ancestor of dogs and humans existed about 95 million years ago and likely had a rather small, simple brain with few higher-order association regions (Kaas 2019). This means that any parallels between canine and human auditory processing would represent a striking example of parallel evolution for adaptation to a shared socio-ecological niche. At the same time, the deep phylogenetic divergence of primate and carnivore brains points toward the likely existence of important differences in the information processing architectures supporting communication. A deeper understanding of these similarities and differences could have value for optimizing interspecies communication in working and service dog functions and for understanding the nature of brain–behavior evolution more broadly.

To conclude, this study provides a validated characterization, quantification, and hierarchical organization of cortico-cortical connections underlying auditory processing in dogs. Our results reveal distributed connectivity between temporal, sensorimotor, premotor, motor, and prefrontal cortices, highlighting two distinct auditory–motor networks. These findings underscore the role of natural and artificial selection in shaping neural networks supporting interspecies interactions, offering a foundation for future research on communication and convergent perception mechanisms in domesticated animals.

## Data Availability

The code used for the analysis and the individual connectivity matrices can be provided upon request for the purpose of validating the analyses published here.

## Annexe 1

**Table:**

ROI	Abbrev.	Full name	Gyri	Lobe	Left volume (mm ^3^ )	Right volume (mm ^3^ )	Index left	Index right	Kosmal nomenclature
1	FRh	Area fissurae orbitalis	Orbital gyrus	Frontal	555	651	31	32	ORB
2	ORBI	Area orbitalis I	Orbital gyrus	Frontal	3353	3170	35	36	ORB
3	ORBII	Area orbitalis II	Orbital gyrus	Frontal	2561	2599	33	34	ORB
4	PGI	Area pregenualis I	Pregenual gyrus	Frontal	150	162	41	42	PG
5	PGII	Area pregenualis II	Pregenual gyrus	Frontal	983	834	39	40	PG
6	PGIII	Area pregenualis III	Pregenual gyrus	Frontal	826	807	37	38	PG
7	POL	Area Polaris	Gyrus proreus	Frontal	784	832	43	44	POL
8	PORD	Area paraorbitalis dorsalis	Orbital gyrus	Frontal	713	743	45	46	PORd
9	PORV	Area paraorbitalis ventralis	Orbital gyrus	Frontal	563	555	47	48	ORB
10	PR	area prorealis	Gyrus proreus	Frontal	457	464	53	54	PR
11	PRLI	Area Prorealis lateralis I	Gyrus proreus	Frontal	459	508	51	52	PRL
12	PRLII	Area prorealis lateralis II	Gyrus proreus	Frontal	2838	3042	49	50	PRL
13	SG	Area subgenualis	Pregenual gyrus	Frontal	737	617	55	56	SG
14	SPRI	Area Subprorealis I	Gyrus subproreus	Frontal	664	617	59	60	SPR
15	SPRII	Area Subprorealis II	Gyrus subproreus	Frontal	1343	1376	57	58	SPR
16	SPRLI	Area subprorealis lateralis I	Gyrus subproreus	Frontal	272	274	63	64	SPRL
17	SPRLII	Area subprorealis Lateralis II	Gyrus subproreus	Frontal	436	495	61	62	SPRL
19	CPM	Area composita medialis I	Posterior compositus gyrus	Temporal	727	844	147	148	CP
20	Eac	Area ectosylvia accessoria	Ectosylvian gyrus	Temporal	2301	2466	151	152	EA
21	EM	Area ectosylvia medialis	Ectosylvian gyrus	Temporal	5434	5741	155	156	EM
22	EPI	Area ectosylvia posterior I	Ectosylvian gyrus	Temporal	2990	3148	157	158	EP
23	SA	Area sylvia	Sylvian gyrus	Temporal	1100	912	manually_divided_SA	manually_divided_SA	SA
24	SD						manually_divided_SD	manually_divided_SD	SD
25	SP						manually_divided_SP	manually_divided_SP	SP
26	C	Area centralis	Central gyrus	Sensory–motor	569	588	183	184	C
27	CA	Area composita anterior	Anterior compositus gyrus	Sensory–motor	2251	2663	171	172	CA
28	CE	Area composita ectosylvia	Anterior compositus gyrus	Sensory–motor	2783	2746	173	174	CE
29	CJ	Area composita interna	Anterior compositus gyrus	Sensory–motor	364	375	175	176	CJ
30	CS	Area composita sigmoid	Anterior compositus gyrus	Sensory–motor	990	1117	179	180	CS
31	CSL	Area composita sigmoidea lateralis	Anterior compositus gyrus	Sensory–motor	1724	1660	177	178	KS
32	CX	Area composita precruciata	Precruciate gyrus	Sensory–motor	365	356	181	182	CX
33	FK	Area fissurae coronalis	Coronal gyrus	Sensory–motor	1419	1469	185	186	KS
34	FPG	Area fissurae pregenualis	Coronal gyrus	Sensory–motor	372	372	187	188	XM
35	FPS	Area fissurae presylviae	Anterior compositus gyrus	Sensory–motor	1345	1375	189	190	FPS
36	FS	Area fissurae splenialis	Precruciate gyrus	Sensory–motor	1038	929	191	192	
37	KA	Area coronalis anterior	Coronal gyrus	Sensory–motor	5189	4987	193	194	KA
38	KM	Area coronalis medialis	Coronal gyrus	Sensory–motor	717	600	195	196	KS
39	PoC	Area postcentralis I	Postcentral gyrus	Sensory–motor	1803	1976	197	198	
40	PrCI/II	Area precentralis I/II	Precentral gyrus	Sensory–motor	1532	1462	199	200	
41	PrCIII	Area precentralis III	Precentral gyrus	Sensory–motor	1124	1230	201	202	
42	PrCJ	Area precentralis interna	Precentral gyrus	Sensory–motor	1244	1246	203	204	
43	PrCL	Area precentral lateralis	Precentral gyrus	Sensory–motor	306	268	205	206	
44	XC	Area precruciata centralis	Precruciate gyrus	Sensory–motor	730	799	207	208	XC
45	XL	Area precruciata lateralis	Precruciate gyrus	Sensory–motor	766	732	209	210	XL
46	XMI	Area precruciata medialis I	Precruciate gyrus	Sensory–motor	642	611	213	214	XM
47	XMII	Area precruciata medialis II	Precruciate gyrus	Sensory–motor	1111	1046	211	212	XM
48	XP	Area precruciata posterior	Precruciate gyrus	Sensory–motor	649	620	215	216	XP
49	MedGen	Medial geniculate	Subcortical regions	Subcortical	333	330	229	230	MGB
